# *N*-(4-Chloro­phen­yl)-2-methyl­benzamide

**DOI:** 10.1107/S1600536809002633

**Published:** 2009-01-28

**Authors:** B. Thimme Gowda, Sabine Foro, B. P. Sowmya, Hiromitsu Terao, Hartmut Fuess

**Affiliations:** a Department of Chemistry, Mangalore University, Mangalagangotri 574 199, Mangalore, India; b Institute of Materials Science, Darmstadt University of Technology, Petersenstrasse 23, D-64287 Darmstadt, Germany; c Faculty of Integrated Arts and Sciences, Tokushima University, Minamijosanjima-cho, Tokushima 770-8502, Japan

## Abstract

In the structure of the title compound, C_14_H_12_ClNO, the N—H and C=O bonds are *trans* to each other. Furthermore, the C=O bond is *syn* to the *ortho*-methyl group in the benzoyl ring, similar to what is observed in 2-methyl-*N*-(4-methyl­phen­yl)benzamide and 2-methyl-*N*-phenyl­benzamide. The amide linkage (–NHCO–) makes dihedral angles of 36.9 (7) and 46.4 (5)° with the aniline and benzoyl rings, respectively, while the dihedral angle between the benzoyl and aniline rings is 83.1 (1)°. In the crystal structure, mol­ecules form chains running along the *b* axis through N—H⋯O hydrogen bonds.

## Related literature

For related structures, see: Gowda *et al.* (2003 ▶, 2008*a* ▶,*b* ▶); Gowda, Tokarčík *et al.* (2008 ▶).
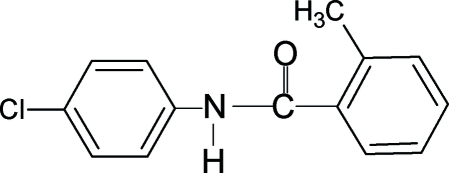


## Experimental

### 

#### Crystal data

C_14_H_12_ClNO
*M* *_r_* = 245.70
Monoclinic, 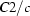

*a* = 22.345 (2) Å
*b* = 5.1092 (4) Å
*c* = 22.222 (1) Å
β = 109.593 (6)°
*V* = 2390.1 (3) Å^3^
*Z* = 8
Cu *K*α radiation
μ = 2.67 mm^−1^
*T* = 299 (2) K
0.50 × 0.13 × 0.13 mm

#### Data collection

Enraf–Nonius CAD-4 diffractometer
Absorption correction: none
2213 measured reflections
2085 independent reflections
1741 reflections with *I* > 2σ(*I*)
*R*_int_ = 0.074
3 standard reflections frequency: 120 min intensity decay: 1.0%

#### Refinement

*R*[*F* ^2^ > 2σ(*F* ^2^)] = 0.046
*wR*(*F* ^2^) = 0.159
*S* = 1.08
2085 reflections
182 parameters
H atoms treated by a mixture of independent and constrained refinement
Δρ_max_ = 0.27 e Å^−3^
Δρ_min_ = −0.39 e Å^−3^

### 

Data collection: *CAD-4-PC* (Enraf–Nonius, 1996 ▶); cell refinement: *CAD-4-PC*; data reduction: *REDU4* (Stoe & Cie, 1987 ▶); program(s) used to solve structure: *SHELXS97* (Sheldrick, 2008 ▶); program(s) used to refine structure: *SHELXL97* (Sheldrick, 2008 ▶); molecular graphics: *PLATON* (Spek, 2003 ▶); software used to prepare material for publication: *SHELXL97*.

## Supplementary Material

Crystal structure: contains datablocks I, global. DOI: 10.1107/S1600536809002633/bt2857sup1.cif

Structure factors: contains datablocks I. DOI: 10.1107/S1600536809002633/bt2857Isup2.hkl

Additional supplementary materials:  crystallographic information; 3D view; checkCIF report

## Figures and Tables

**Table 1:** Hydrogen-bond geometry (Å, °)

*D*—H⋯*A*	*D*—H	H⋯*A*	*D*⋯*A*	*D*—H⋯*A*
N1—H1N⋯O1^i^	0.84 (3)	2.14 (3)	2.937 (3)	159 (2)

Symmetry code: (i) 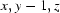.

**Fig. 1.**
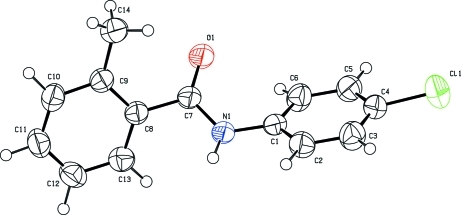
Molecular structure of the title compound, showing the atom labeling scheme. The displacement ellipsoids are drawn at the 50% probability level. The H atoms are represented as small spheres of arbitrary radii.

**Fig. 2.**
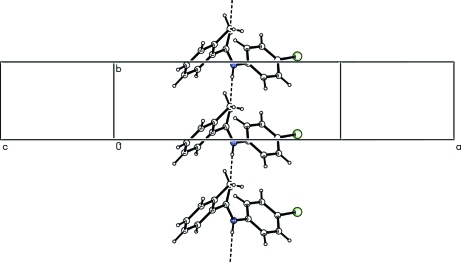
Molecular packing of the title compound with hydrogen bonding shown as dashed lines..

## supplementary crystallographic information

### Comment

In the present work, as part of a study of the substituent effects on the solid state structures of benzanilides (Gowda *et al.*, 2003; 2008*a, b, c*), the structure of 2-methyl-*N*-(4-chlorophenyl)- benzamide has been determined. In the structure of the title compound (Fig. 1), the N—H and C=O bonds are *trans* to each other. Further, the   C=O bond is *syn* to the *ortho*-methyl substituent in the benzoyl ring. These observations are similar to those observed in 2-methyl-*N*-(phenyl)-benzamide (Gowda *et al.*, 2008*a*), 2-methyl-*N*-(4-methylphenyl)- benzamide   (Gowda, Tokarčík *et al.*, 2008), 2-methyl-*N*- (2-chlorophenyl)-benzamide   and 2-methyl-*N*-(3-chlorophenyl)- benzamide  (Gowda *et al.*, 2008*b*). The amide linkage, –NHCO– makes   dihedral angles of 36.9 (7)° and 46.4 (5)° with the aniline and benzoyl rings, respectively, while the dihedral angle between the benzoyl and aniline rings is 83.1 (1)°, in comparison with the central amide group –NHCO– being tilted to the benzoyl ring at an angle of 60.0 (1)° and the two rings (benzoyl & aniline) making a dihedral angle of 81.4 (1)° in N4MP2MBA. The other bond parameters in the title compound are similar to those in the previously mentioned structures. The packing diagram   shows N—H···O (Table 1)  hydrogen bonds connnecting the molecules into chains running along the b-axis (Fig. 2).

### Experimental

The title compound was prepared according to the literature method (Gowda *et al.*, 2003). The purity of the compound was checked by determining its melting point. It was characterized by recording its infrared and NMR spectra. Single crystals of the title compound used in X-ray diffraction studies were obtained from a slow evaporation of its ethanolic solution at room temperature.

### Refinement

The H atoms of the methyl group were positioned with idealized geometry using a riding model with C—H = 0.96 Å. The other H atoms were located in difference map, and their positional parameters were refined freely. The isotropic displacement parameters of all H atoms were set to 1.2 *U*_eq_(C-aromatic, N) or 1.5 *U*_eq_(C-methyl).

### Crystal data

**Table:**

C_14_H_12_ClNO	*F*(000) = 1024
*M**_r_* = 245.70	*D*_x_ = 1.366 Mg m^−^^3^
Monoclinic, *C*2/*c*	Cu *K*α radiation, λ = 1.54180 Å
Hall symbol: -C 2yc	Cell parameters from 25 reflections
*a* = 22.345 (2) Å	θ = 8.1–22.2°
*b* = 5.1092 (4) Å	µ = 2.67 mm^−^^1^
*c* = 22.222 (1) Å	*T* = 299 K
β = 109.593 (6)°	Rod, colourless
*V* = 2390.1 (3) Å^3^	0.50 × 0.13 × 0.13 mm
*Z* = 8	

### Data collection

**Table:**

Enraf–Nonius CAD-4 diffractometer	*R*_int_ = 0.074
Radiation source: fine-focus sealed tube	θ_max_ = 66.9°, θ_min_ = 4.2°
graphite	*h* = −25→26
ω/2θ scans	*k* = −6→0
2213 measured reflections	*l* = −26→1
2085 independent reflections	3 standard reflections every 120 min
1741 reflections with *I* > 2σ(*I*)	intensity decay: 1.0%

### Refinement

**Table:**

Refinement on *F*^2^	Secondary atom site location: difference Fourier map
Least-squares matrix: full	Hydrogen site location: inferred from neighbouring sites
*R*[*F*^2^ > 2σ(*F*^2^)] = 0.046	H atoms treated by a mixture of independent and constrained refinement
*wR*(*F*^2^) = 0.159	*w* = 1/[σ^2^(*F*_o_^2^) + (0.1089*P*)^2^ + 0.7738*P*] where *P* = (*F*_o_^2^ + 2*F*_c_^2^)/3
*S* = 1.08	(Δ/σ)_max_ = 0.002
2085 reflections	Δρ_max_ = 0.27 e Å^−^^3^
182 parameters	Δρ_min_ = −0.39 e Å^−^^3^
0 restraints	Extinction correction: *SHELXL97* (Sheldrick, 2008), Fc^*^=kFc[1+0.001xFc^2^λ^3^/sin(2θ)]^-1/4^
Primary atom site location: structure-invariant direct methods	Extinction coefficient: 0.0012 (3)

### Special details

**Table:**

Geometry. All e.s.d.'s (except the e.s.d. in the dihedral angle between two l.s. planes) are estimated using the full covariance matrix. The cell e.s.d.'s are taken into account individually in the estimation of e.s.d.'s in distances, angles and torsion angles; correlations between e.s.d.'s in cell parameters are only used when they are defined by crystal symmetry. An approximate (isotropic) treatment of cell e.s.d.'s is used for estimating e.s.d.'s involving l.s. planes.
Refinement. Refinement of *F*^2^ against ALL reflections. The weighted *R*-factor *wR* and goodness of fit *S* are based on *F*^2^, conventional *R*-factors *R* are based on *F*, with *F* set to zero for negative *F*^2^. The threshold expression of *F*^2^ > σ(*F*^2^) is used only for calculating *R*-factors(gt) *etc*. and is not relevant to the choice of reflections for refinement. *R*-factors based on *F*^2^ are statistically about twice as large as those based on *F*, and *R*- factors based on ALL data will be even larger.

### Fractional atomic coordinates and isotropic or equivalent isotropic displacement parameters (Å^2^)

**Table:**

	*x*	*y*	*z*	*U*_iso_*/*U*_eq_	
C1	0.38579 (10)	−0.0174 (4)	0.97284 (10)	0.0427 (5)	
C2	0.43053 (12)	−0.1978 (5)	0.96925 (11)	0.0531 (6)	
H2	0.4467 (13)	−0.343 (6)	1.0021 (12)	0.064*	
C3	0.45905 (12)	−0.1751 (6)	0.92298 (12)	0.0582 (6)	
H3	0.4906 (14)	−0.296 (6)	0.9225 (13)	0.070*	
C4	0.44190 (11)	0.0270 (5)	0.88024 (10)	0.0501 (6)	
C5	0.39547 (13)	0.2001 (5)	0.88127 (11)	0.0556 (6)	
H5	0.3833 (13)	0.344 (7)	0.8502 (13)	0.067*	
C6	0.36711 (12)	0.1774 (5)	0.92743 (10)	0.0541 (6)	
H6	0.3328 (14)	0.277 (6)	0.9283 (12)	0.065*	
C7	0.34840 (11)	0.1669 (4)	1.05601 (10)	0.0464 (5)	
C8	0.32646 (10)	0.0953 (4)	1.11029 (10)	0.0422 (5)	
C9	0.35181 (9)	0.2246 (4)	1.16938 (9)	0.0437 (5)	
C10	0.32915 (11)	0.1468 (5)	1.21792 (11)	0.0529 (6)	
H10	0.3491 (12)	0.242 (6)	1.2606 (13)	0.063*	
C11	0.28353 (12)	−0.0416 (5)	1.20908 (12)	0.0573 (6)	
H11	0.2695 (14)	−0.099 (6)	1.2430 (14)	0.069*	
C12	0.25847 (11)	−0.1652 (5)	1.15110 (12)	0.0567 (6)	
H12	0.2257 (13)	−0.308 (6)	1.1438 (12)	0.068*	
C13	0.28029 (11)	−0.0955 (5)	1.10188 (11)	0.0497 (5)	
H13	0.2621 (12)	−0.182 (6)	1.0597 (12)	0.060*	
C14	0.40270 (12)	0.4269 (5)	1.18350 (12)	0.0570 (6)	
H14A	0.4283	0.4180	1.2278	0.068*	
H14B	0.3837	0.5973	1.1741	0.068*	
H14C	0.4288	0.3958	1.1576	0.068*	
N1	0.36006 (10)	−0.0392 (4)	1.02268 (8)	0.0461 (5)	
H1N	0.3609 (12)	−0.189 (6)	1.0382 (12)	0.055*	
O1	0.35554 (12)	0.3935 (3)	1.04201 (9)	0.0740 (6)	
Cl1	0.48134 (3)	0.07146 (16)	0.82524 (3)	0.0760 (3)	

### Atomic displacement parameters (Å^2^)

**Table:**

	*U*^11^	*U*^22^	*U*^33^	*U*^12^	*U*^13^	*U*^23^
C1	0.0526 (11)	0.0351 (10)	0.0411 (10)	−0.0047 (9)	0.0164 (9)	−0.0033 (8)
C2	0.0640 (13)	0.0443 (13)	0.0541 (12)	0.0073 (10)	0.0240 (10)	0.0077 (10)
C3	0.0597 (13)	0.0582 (15)	0.0617 (14)	0.0086 (11)	0.0270 (11)	0.0013 (12)
C4	0.0588 (12)	0.0507 (13)	0.0445 (11)	−0.0110 (10)	0.0220 (10)	−0.0055 (9)
C5	0.0773 (15)	0.0458 (14)	0.0448 (11)	0.0035 (11)	0.0220 (10)	0.0053 (10)
C6	0.0688 (14)	0.0485 (14)	0.0479 (12)	0.0123 (11)	0.0233 (10)	0.0040 (10)
C7	0.0638 (12)	0.0334 (11)	0.0459 (11)	0.0005 (9)	0.0234 (9)	0.0011 (8)
C8	0.0485 (10)	0.0354 (11)	0.0447 (10)	0.0052 (8)	0.0185 (8)	0.0017 (8)
C9	0.0458 (10)	0.0394 (11)	0.0479 (11)	0.0053 (8)	0.0184 (8)	0.0003 (8)
C10	0.0592 (13)	0.0557 (14)	0.0485 (11)	0.0053 (11)	0.0245 (10)	−0.0031 (10)
C11	0.0628 (14)	0.0616 (15)	0.0590 (13)	0.0013 (11)	0.0358 (12)	0.0055 (11)
C12	0.0522 (12)	0.0537 (14)	0.0695 (15)	−0.0074 (11)	0.0274 (11)	0.0023 (12)
C13	0.0513 (11)	0.0461 (13)	0.0505 (11)	−0.0004 (10)	0.0155 (9)	0.0001 (10)
C14	0.0600 (13)	0.0505 (15)	0.0621 (13)	−0.0057 (10)	0.0226 (11)	−0.0080 (10)
N1	0.0669 (11)	0.0315 (9)	0.0455 (10)	0.0002 (8)	0.0262 (8)	0.0018 (7)
O1	0.1407 (18)	0.0319 (9)	0.0708 (11)	−0.0004 (10)	0.0636 (12)	0.0022 (7)
Cl1	0.0838 (5)	0.0892 (6)	0.0706 (5)	−0.0083 (4)	0.0465 (4)	0.0039 (4)

### Geometric parameters (Å, °)

**Table:**

C1—C6	1.378 (3)	C8—C13	1.386 (3)
C1—C2	1.382 (3)	C8—C9	1.408 (3)
C1—N1	1.413 (3)	C9—C10	1.394 (3)
C2—C3	1.384 (3)	C9—C14	1.490 (3)
C2—H2	1.02 (3)	C10—C11	1.368 (4)
C3—C4	1.368 (4)	C10—H10	1.03 (3)
C3—H3	0.94 (3)	C11—C12	1.374 (4)
C4—C5	1.369 (4)	C11—H11	0.95 (3)
C4—Cl1	1.745 (2)	C12—C13	1.385 (3)
C5—C6	1.380 (3)	C12—H12	1.01 (3)
C5—H5	0.98 (3)	C13—H13	0.99 (3)
C6—H6	0.93 (3)	C14—H14A	0.9600
C7—O1	1.223 (3)	C14—H14B	0.9600
C7—N1	1.362 (3)	C14—H14C	0.9600
C7—C8	1.492 (3)	N1—H1N	0.84 (3)
			
C6—C1—C2	119.1 (2)	C10—C9—C8	116.8 (2)
C6—C1—N1	121.9 (2)	C10—C9—C14	119.0 (2)
C2—C1—N1	119.03 (19)	C8—C9—C14	124.18 (19)
C1—C2—C3	120.6 (2)	C11—C10—C9	122.4 (2)
C1—C2—H2	122.3 (15)	C11—C10—H10	122.6 (16)
C3—C2—H2	116.9 (15)	C9—C10—H10	115.0 (16)
C4—C3—C2	119.2 (2)	C10—C11—C12	120.5 (2)
C4—C3—H3	121.7 (17)	C10—C11—H11	122.0 (18)
C2—C3—H3	119.1 (17)	C12—C11—H11	117.4 (19)
C3—C4—C5	120.9 (2)	C11—C12—C13	118.8 (2)
C3—C4—Cl1	119.67 (19)	C11—C12—H12	122.0 (15)
C5—C4—Cl1	119.40 (19)	C13—C12—H12	119.2 (15)
C4—C5—C6	119.8 (2)	C12—C13—C8	121.2 (2)
C4—C5—H5	120.3 (17)	C12—C13—H13	119.4 (16)
C6—C5—H5	119.8 (17)	C8—C13—H13	119.4 (16)
C1—C6—C5	120.2 (2)	C9—C14—H14A	109.5
C1—C6—H6	115.6 (17)	C9—C14—H14B	109.5
C5—C6—H6	124.0 (17)	H14A—C14—H14B	109.5
O1—C7—N1	121.9 (2)	C9—C14—H14C	109.5
O1—C7—C8	122.95 (19)	H14A—C14—H14C	109.5
N1—C7—C8	115.14 (19)	H14B—C14—H14C	109.5
C13—C8—C9	120.3 (2)	C7—N1—C1	124.59 (19)
C13—C8—C7	119.68 (19)	C7—N1—H1N	117.7 (18)
C9—C8—C7	120.03 (19)	C1—N1—H1N	115.8 (18)
			
C6—C1—C2—C3	3.7 (4)	C7—C8—C9—C10	−179.84 (19)
N1—C1—C2—C3	−176.8 (2)	C13—C8—C9—C14	178.3 (2)
C1—C2—C3—C4	−0.7 (4)	C7—C8—C9—C14	−2.8 (3)
C2—C3—C4—C5	−2.4 (4)	C8—C9—C10—C11	−1.4 (3)
C2—C3—C4—Cl1	175.54 (19)	C14—C9—C10—C11	−178.6 (2)
C3—C4—C5—C6	2.5 (4)	C9—C10—C11—C12	0.7 (4)
Cl1—C4—C5—C6	−175.48 (19)	C10—C11—C12—C13	0.0 (4)
C2—C1—C6—C5	−3.7 (4)	C11—C12—C13—C8	−0.1 (4)
N1—C1—C6—C5	176.9 (2)	C9—C8—C13—C12	−0.6 (3)
C4—C5—C6—C1	0.6 (4)	C7—C8—C13—C12	−179.5 (2)
O1—C7—C8—C13	135.1 (3)	O1—C7—N1—C1	5.2 (4)
N1—C7—C8—C13	−44.8 (3)	C8—C7—N1—C1	−174.86 (18)
O1—C7—C8—C9	−43.7 (3)	C6—C1—N1—C7	−41.2 (3)
N1—C7—C8—C9	136.3 (2)	C2—C1—N1—C7	139.3 (2)
C13—C8—C9—C10	1.3 (3)		

### Hydrogen-bond geometry (Å, °)

**Table:**

*D*—H···*A*	*D*—H	H···*A*	*D*···*A*	*D*—H···*A*
N1—H1N···O1^i^	0.84 (3)	2.14 (3)	2.937 (3)	159 (2)

Symmetry codes: (i) *x*, *y*−1, *z*.
